# Trends in the Use of Telehealth During the Emergence of the COVID-19 Pandemic — United States, January–March 2020

**DOI:** 10.15585/mmwr.mm6943a3

**Published:** 2020-10-30

**Authors:** Lisa M. Koonin, Brooke Hoots, Clarisse A. Tsang, Zanie Leroy, Kevin Farris, Brandon Jolly, Peter Antall, Bridget McCabe, Cynthia B.R. Zelis, Ian Tong, Aaron M. Harris

**Affiliations:** ^1^Healthcare Systems and Worker Safety Task Force, CDC COVID-19 Emergency Response Team; ^2^HHS COVID-19 Health Care Resilience Task Force; ^3^Amwell Medical Group, Boston, Massachusetts; ^4^Teladoc Health, Inc., Purchase, New York; ^5^MDLIVE, Miramar, Florida; ^6^Doctor on Demand, Inc., San Francisco, California.

In February 2020, CDC issued guidance advising persons and health care providers in areas affected by the coronavirus disease 2019 (COVID-19) pandemic to adopt social distancing practices, specifically recommending that health care facilities and providers offer clinical services through virtual means such as telehealth.* Telehealth is the use of two-way telecommunications technologies to provide clinical health care through a variety of remote methods.^†^ To examine changes in the frequency of use of telehealth services during the early pandemic period, CDC analyzed deidentified encounter (i.e., visit) data from four of the largest U.S. telehealth providers that offer services in all states.^§^ Trends in telehealth encounters during January–March 2020 (surveillance weeks 1–13) were compared with encounters occurring during the same weeks in 2019. During the first quarter of 2020, the number of telehealth visits increased by 50%, compared with the same period in 2019, with a 154% increase in visits noted in surveillance week 13 in 2020, compared with the same period in 2019. During January–March 2020, most encounters were from patients seeking care for conditions other than COVID-19. However, the proportion of COVID-19–related encounters significantly increased (from 5.5% to 16.2%; p<0.05) during the last 3 weeks of March 2020 (surveillance weeks 11–13). This marked shift in practice patterns has implications for immediate response efforts and longer-term population health. Continuing telehealth policy changes and regulatory waivers might provide increased access to acute, chronic, primary, and specialty care during and after the pandemic.

Data for this analysis were provided to CDC from four large national telehealth providers as part of partner engagement to monitor and improve outcomes during the COVID-19 pandemic. Datasets included the date of the telehealth encounter, patient sex, age, county and state of residence, and, for 2020 visits, disposition after the visit (e.g., home or location the provider recommended that the patient seek additional care, if needed, such as in an emergency department [ED] or with a primary care provider), “reason for visit” (text field), and diagnosis defined by one or more *International Classification of Diseases, Tenth Revision* (ICD-10) codes.^¶^ No patient, facility, or provider identifiers were included in the datasets. Date of encounter was categorized by epidemiologic surveillance week. For comparison, total ED visit volume by surveillance week in 2019 and 2020 was analyzed from National Syndromic Surveillance Program (NSSP) data, and percentage change from 2019 to 2020 was calculated by week. The national data in NSSP includes ED visits from a subset of hospitals in 47 states, accounting for approximately 73% of ED visits in the United States.

 Patient encounters for 2020 were characterized as COVID-19–related or not COVID-19–related. COVID-19–related visits were defined as those with one or more of the following: 1) signs and symptoms in the “reason for visit” field meeting criteria established by CDC in March 2020 for COVID-19–like illness,** 2) ICD-10 codes in the diagnosis field for Z20.828 (contact with and suspected exposure to other viral communicable diseases) or U07.1 (2019-nCoV acute respiratory disease), or 3) the terms “COVID” or “coronavirus” in the “reason for visit” field. COVID-19–like illness was defined as fever plus cough or sore throat or shortness of breath. Patient encounters that did not include one of the described criteria were categorized as not COVID-19–related. This activity was reviewed by CDC and was conducted consistent with applicable federal law and CDC policy: [45 C.F.R. part 46.102(l)(2); 21 C.F.R. part 56; 42 U.S.C. Sect. 241(d); 5 U.S.C. Sect. 552a; 44 U.S.C. Sect. 3501, et seq.]

A Wilcoxon signed-rank test was used to test the difference in the median encounter count by week from 2019 to 2020. Average weekly percent changes in encounter count were calculated using Joinpoint Regression Analysis Software (version 4.8.0.1).^††^ Pairwise comparisons of proportions of encounters between weeks were calculated with chi-squared tests; p values <0.05 were considered statistically significant. Approximately 2.7 million encounter records were available for analysis. Approximately 1,629,000 telehealth encounters occurred in the first 3 months of 2020 (early pandemic period), compared with approximately 1,084,000 encounters during the same period in 2019 (50% increase overall; p<0.05). During surveillance week 13 in 2020, telehealth visits increased 154% (p<0.05), compared with the same week in 2019 (Figure 1). In contrast, the number of ED visits in the last 3 weeks of March 2020 decreased markedly, compared with the same period in 2019.

**FIGURE 1:**
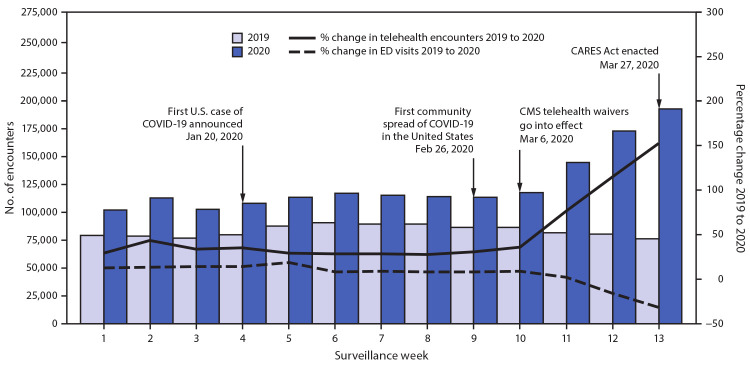
Number of telehealth patient encounters reported by four telehealth providers that offer services in all states and percentage change in telehealth encounters and emergency department (ED) visits — United States, January 1–March 30, 2019 (comparison period) and January 1–March 28, 2020 (early pandemic period)* **Abbreviations:** CARES Act = Coronavirus Aid, Relief, and Economic Security Act; CMS = Center for Medicare & Medicaid Services; COVID-19 = coronavirus disease 2019. * Unpublished ED visit data obtained from the National Syndromic Surveillance Program.

Most telehealth encounters were for adults aged 18–49 years (66% in 2019 and 69% in 2020) and female patients (63% in both 2019 and 2020). During the early pandemic period in 2020, the percentage of telehealth visits for persons aged 18–49 years increased slightly, from 68% during the first week of January 2020 to 73% during the last week of March (p<0.05). There was a slight decrease in the percentage of telehealth encounters for children during the emerging pandemic period, compared with the same period in 2019. An average of 3.5% of encounters were for children aged <5 years in 2020 (compared with 4.0% in 2019), and 8.6% were for those aged 5–17 years in 2020 (compared with 10.0% in 2019).

During January–March 2020, most telehealth patients (93%) sought care for conditions other than COVID-19. However, the proportion of COVID-19–related encounters grew (from 5.5% to 16.2%; p<0.05) during the last 3 weeks of March, when an increasing number of visits included mention of COVID-19 in the “reason for visit” field (Figure 2). In addition, 69% of patients who had a telehealth encounter during the early pandemic period in 2020 were managed at home, with 26% advised to seek follow-up from their primary care provider as needed or, if their condition worsened or did not improve, 1.5% were advised to seek care in an ED, and 3% were referred to an urgent care setting. During 2020, referral patterns were consistent during the early pandemic period; the increases or decreases in referral categories between weeks 1–9 and weeks 10–13 were <1%.

**FIGURE 2:**
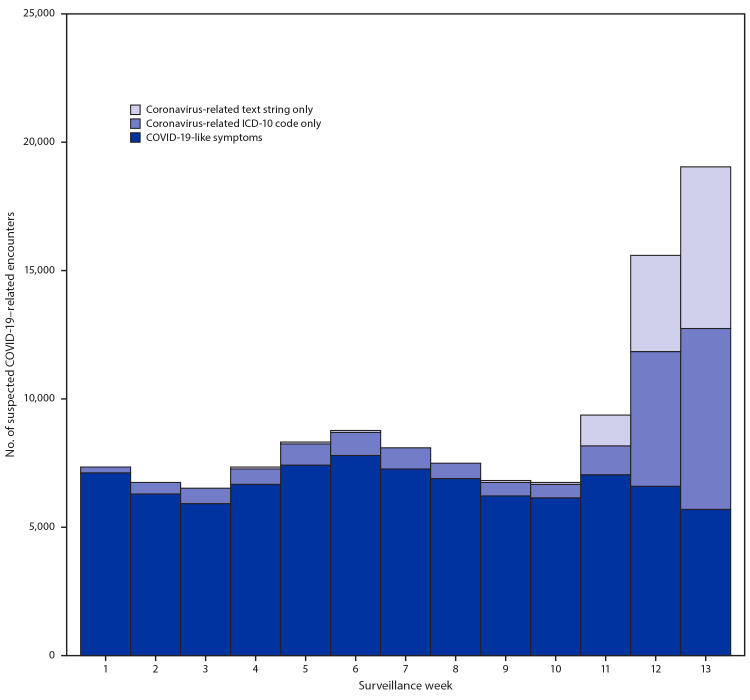
Number of telehealth patient encounters for persons with COVID-19-like symptoms, coronavirus-related ICD-10 codes, or coronavirus-related text string entries reported by four telehealth providers that offer services in all states — United States, January 1–March 28, 2020 **Abbreviations: **COVID-19 = coronavirus disease 2019; ICD-10 = *International Classification of Diseases, Tenth Revision*.

## Discussion

This cross-sectional analysis of telehealth use during the emergence of the COVID-19 pandemic in the United States (January–March 2020) provides information on use patterns of this health care delivery modality for planners and providers. The age and sex of patients who accessed telehealth services in this analysis were similar to those seeking telehealth services in other studies (*1*). Substantially more telehealth visits were made during the first 3 months of 2020 than during the same period in 2019; whereas visits to EDs sharply declined. Other researchers have noted a marked overall increase in the use of telehealth services in the latter weeks of March 2020 and sharp declines in the use of EDs (*2*–*4*). Overall, an estimated 41%–42% of U.S. adults reported having delayed or avoided seeking care during the pandemic because of concerns about COVID-19, including 12% who reported having avoided seeking urgent or emergency care (*3*,*4*). The sharp rise in telehealth encounters might be temporally associated with these declines in in-person visits. The increased number of visits in the latter weeks in March, 2020 might also be related to the March 6, 2020 policy changes and regulatory waivers from Centers for Medicare & Medicaid Services^§§^ (1,135 waivers) in response to COVID-19 and provisions of the U.S. Coronavirus Aid, Relief, and Economic Security (CARES) Act, effective March 27, 2020.^¶¶^ These emergency policies included improved provider payments for telehealth, allowance for providers to serve out-of-state patients, authorization for multiple types of providers to offer telehealth services, reduced or waived cost-sharing for patients, and permission for federally qualified health centers or rural health clinics to offer telehealth services. The waivers also allowed for virtual visits to be conducted from the patient’s home, rather than in a health care setting. Other contributing factors that could have affected utilization of services include state-issued stay-at-home orders (*5*), states’ inclusion of telehealth as a Medicaid covered benefit,*** and CDC’s guidance for social distancing and increased use of virtual clinical visits.

Telehealth might have multiple benefits for public and individual health during the COVID-19 pandemic. During the latter weeks in March 2020, remote screening and management of persons who needed clinical care for COVID-19 and other conditions might have increased access to care when many outpatient offices were closed or had limited operating hours. The increased availability of telehealth services also might have reduced disease exposure for staff members and patients, preserved scarce supplies of personal protective equipment, and minimized patient surge on facilities (*6*). In addition, most patients seeking telehealth in the early pandemic period were managed at home, which might have reduced large volumes of patients seeking care at health care facilities. Access to telehealth services might have been particularly valuable for those patients who were reluctant to seek in-person care, had difficulty accessing in-person care or who had chronic conditions that place them at high risk for severe COVID-19 (*1*).

Although telehealth is generally well-accepted by patients and clinicians (*7*), it is not without challenges. Limited access to the Internet or devices such as smartphones, tablets, or computers, and lack of familiarity with technology might be potential barriers for some patients (*1*,*8*). In addition, virtual visits might not be appropriate for some persons based on level of acuity or necessity to conduct an in-person physical examination or diagnostic testing. Although several reports have described concern in the decline of emergency department use during the early pandemic period, a very small proportion of telehealth patients in this analysis were referred to emergency care. Increases in the use of telehealth precipitated by COVID could have long-term benefits for improving appropriate emergency department utilization. 

The findings in this report are subject to at least two limitations. First, the data in this analysis are from a sample of four large national telehealth providers and do not represent all virtual encounters conducted during the study period. In addition, the symptoms used initially to identify patients with possible COVID-19 were limited, and it was not possible to distinguish them from those with influenza-like illness symptoms or other respiratory conditions; therefore, some patients might have been unidentified or misclassified.

Health care delivery has shifted during the COVID-19 pandemic, with telehealth encounters sharply increasing in late March 2020. Telehealth can serve an important role in pandemic planning and response. Continued availability and promotion of telehealth services might play a prominent role in increasing access to services during the public health emergency. The regulatory waivers in place during COVID-19 might have helped increase adoption of telehealth services along with public health guidance encouraging virtual visits and CDC recommendations for use of telehealth services during the COVID-19 pandemic.^†††^ Data from telehealth encounters can inform public health surveillance systems, especially during the pandemic. With expanded access and improved reimbursement policies in place, as well as ongoing acceptability by patients and health care providers, telehealth might continue to serve as an important modality for delivering care during and after the pandemic.^§§§^

**Summary**

**What is already known about this topic?**

Use of telehealth (the remote provision of clinical care) early during the COVID-19 pandemic has not been well characterized.

**What is added by this report?**

The 154% increase in telehealth visits during the last week of March 2020, compared with the same period in 2019 might have been related to pandemic-related telehealth policy changes and public health guidance.

**What are the implications for public health practice?**

Telehealth could have multiple benefits during the pandemic by expanding access to care, reducing disease exposure for staff and patients, preserving scarce supplies of personal protective equipment, and reducing patient demand on facilities. Telehealth policy changes might continue to support increased care access during and after the pandemic.

## Footnotes

* https://www.cdc.gov/coronavirus/2019-ncov/hcp/guidance-hcf.html.

† https://telehealth.hhs.gov/patients/understanding-telehealth/#what-is-telehealth.

§ Amwell Medical Group, Boston, Massachusetts; Teladoc Health, Inc., Purchase, New York; MDLIVE, Miramar, Florida; and Doctor on Demand, Inc., San Francisco, California.

¶ https://www.cdc.gov/nchs/icd/icd10cm.htm.

** Symptoms used to characterize COVID-19–like illness during January–March 2020 included fever, cough and shortness of breath https://www.cdc.gov/nchs/data/icd/interim-coding-advice-coronavirus-March-2020-final.pdf. Since that time, CDC has expanded the list of symptoms associated with this illness. https://www.cdc.gov/coronavirus/2019-ncov/symptoms-testing/symptoms.html.

†† https://surveillance.cancer.gov/joinpoint/.

§§ https://www.cms.gov/newsroom/fact-sheets/medicare-telemedicine-health-care-provider-fact-sheet.

¶¶ https://www.congress.gov/bill/116th-congress/senate-bill/3548/text?q=product+update.

*** https://www.medicaid.gov/medicaid/benefits/telemedicine/index.html.

††† https://www.cdc.gov/coronavirus/2019-ncov/hcp/telehealth.html.

§§§ https://www.federalregister.gov/d/2020-17364/improving-rural-health-and-telehealth-access.
