# *Erratum:* Vol. 69, No. 43

**DOI:** 10.15585/mmwr.mm6945a9

**Published:** 2020-11-13

In the report “Trends in the Use of Telehealth During the Emergence of the COVID-19 Pandemic — United States, January–March 2020,” on page 1595, a name in the list of authors was incorrect. The author’s name should have read **B. Tilman Jolly**. In addition, on page 1597, a comma was mistakenly included in the phrase 1,135 waivers. The phrase should have read “**1135** waivers.”

